# Positive Attributes of Aging and Connections to Health: Evidence From the National Poll on Healthy Aging

**DOI:** 10.1093/geroni/igaa057.2557

**Published:** 2020-12-16

**Authors:** Erica Solway, Julie Ober Allen, Matthias Kirch, Dianne Singer, Jeffrey Kullgren, Preeti Malani

**Affiliations:** 1 University of Michigan, Ann Arbor, Michigan, United States; 2 University of Michigan Medical School, Ann Arbor, Michigan, United States

## Abstract

This study explored the prevalence of positive attributes of aging among older adults in a nationally representative sample age 50-80 ((N=2,048, 52% female, 71% White). Nearly 70% of older adults reported that people sought their guidance because of their wisdom and experience. Older adults reported that, as they have gotten older, they have become more comfortable with themselves (88%), have a strong sense of purpose (80%), feel more positively about aging (67%), and have found their life to be better than they had thought it would be (65%). Over half (52%) of those who said their lives were better than they thought reported very good or excellent physical health. Among those who disagreed, only one out of four (25%) reported very good/excellent physical health; similar results were found for mental health (48% vs. 22%). This session will describe positive attributes of aging and their association to physical and mental health.

